# Supplementary material for: Förster Resonance Energy Transfer Measurements in Living Bacteria for Interaction Studies of BamA with BamD and Inhibitor Identification
Source: Cells. 2024 Nov 8;13(22):1858. doi: 10.3390/cells13221858 (PMC11592675; doi:10.3390/cells13221858)
Supplement: Supplementary file 1 [file cells-13-01858-s001.zip › cells-3288926-supplementary.pdf]

Supplementary information

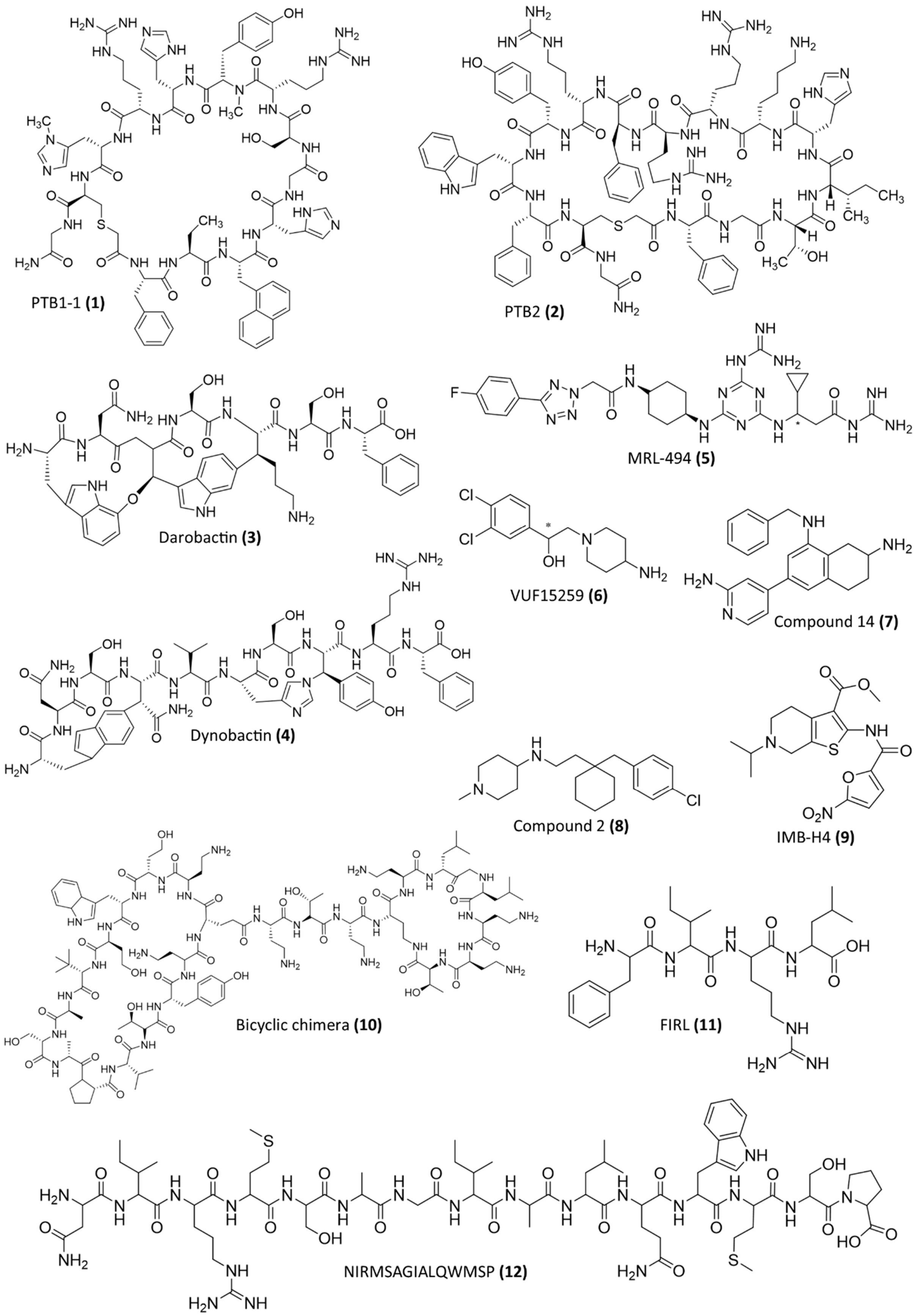

Supplementary Figure S1. Structures of reported BamA inhibitors: PTB1-1 (1) and PTB2 (2) [14], Darobactin (3) [22], Dynobactin (4) [30], MRL-494 (5) [50], VUF15259 (6) [55], Compound 14 (7) and Compound 2 (8) [56] and (12) [26], BamA – BamD interaction inhibitors: IMB-H4 (9) [31], FIRL (11) [30] and molecules disrupting the outer membrane that bind to BamA: Bicyclic chimera (10) [31].

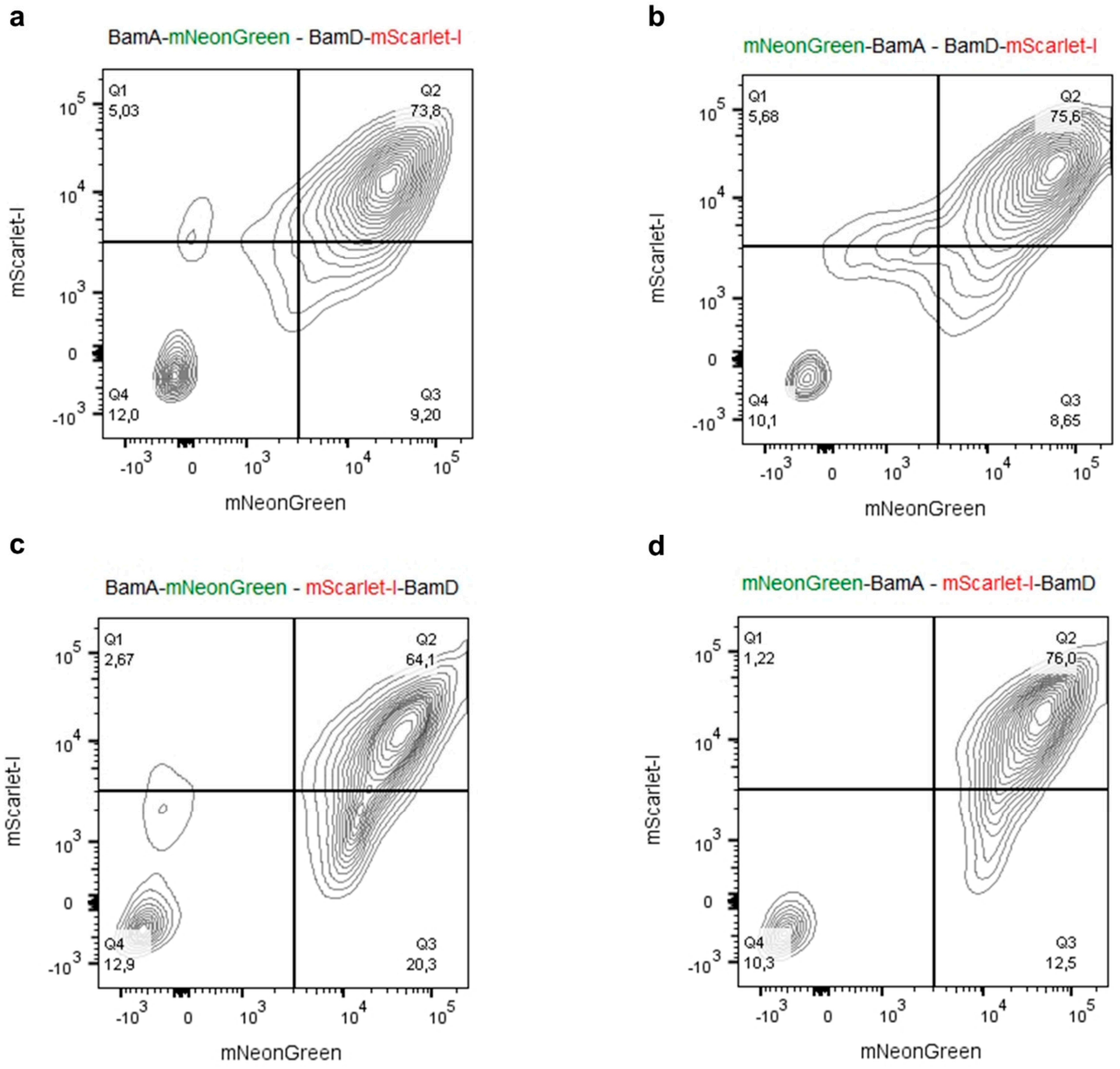

Supplementary Figure S2. Contour plots of cells expressing fluorescently labeled BamA and BamD. Cells were grown as described in materials and methods and subsequently analyzed by flow cytometry. The intensity in the mNeonGreen (donor) channel is plotted against the intensity in the mScarlet-I (acceptor) channel. 50,000 cells inside Q2 were analyzed per sample. **(a)** Cells expressing the BamA-mNeonGreen – BamD-mScarlet-I FRET pair, **(b)** the mNeonGreen-BamA – BamD-mScarlet-I FRET pair, **(c)** the BamA-mNeonGreen – mScarlet-I-BamD FRET pair and **(d)** the mNeonGreen-BamA – mScarlet-I-BamD FRET pair.

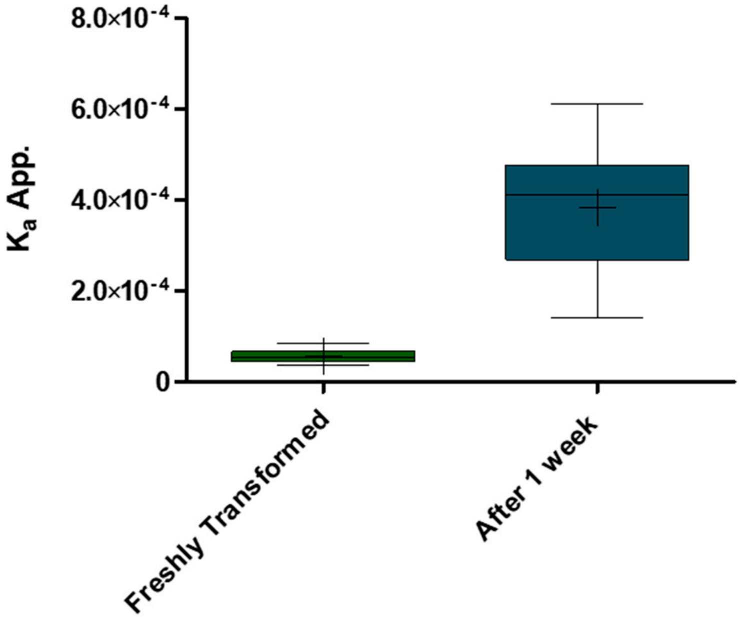

Supplementary Figure S3. Apparent binding affinities ($K_a^{app.}$) of the interaction between BamA and BamD determined with different the BamA-mNeonGreen – BamD-mScarlet-I FRET pair in living cells by fitting a binding model to normalized FRET values. Cells were either directly transformed two days before the experiment (Freshly Transformed) or transformed one week prior to the experiment and kept on an agar plate at 4°C (After 1 week). In both cases overnight and main cultures were prepared as described in *2.3*.

Table S1. Plasmids used in this study

Number	Name	Determinants
pSN03	mNeonGreen-mScarlet-I	KanR, pBBR1 ori, coding for a mNeonGreen-G_4S-mScarlet-I fusion protein under control of an araBAD promotor
pSN08	mScarlet-I-BamD	Bla, pBR322 ori, coding for a SP_{BamD}-mScar-G_4S-BamD fusion protein under control of a Rha promotor
pSN09	BamD-mScarlet-I	Bla, pBR322 ori, coding for a SP_{BamD}-BamD-G_4S-mScar fusion protein under control of a Rha promotor
pSN43	BamA-mNeonGreen – mScarlet-I	KanR, pBBR1 ori, coding for a BamA-mScarlet-I fusion protein connected covalently by a G_4S-linker und control of an araBAD promotor, conding for mNeon under control of a Rha promotor
pSN76	mNeonGreen-BamA	KanR, pBBR1 ori, coding for a SP_{BamA}-mNeon-G_4S-BamA fusion protein under control of an araBAD promotor
pSN77	BamA-mNeonGreen	KanR, pBBR1 ori, coding for a SP_{BamA}-BamA-G_4S-mNeon fusion protein under control of an araBAD promotor
pSN101	mNeonGreen-$BamA^{E373K}$ – mScarlet-I-BamD	Based on pSN142, coding for the $BamA^{E373K}$ variant
pSN103	$BamA^{E373K}$-mNeon – mScarlet-I-BamD	Based on pSN144, coding for the $BamA^{E373K}$ variant
pSN141	mNeonGreen-BamA – BamD-mScarlet-I	KanR, pBBR1 ori, coding for a SP_{BamA}-mNeon-G_4S-BamA fusion protein under control of an araBAD promotor, coding for a SP_{BamD}-BamD-G_4S-mScar fusion protein under control of a Rha promotor
pSN142	mNeonGreen-BamA – mScarlet-I-BamD	KanR, pBBR1 ori, coding for a SP_{BamA}-mNeon-G_4S-BamA fusion protein under control of an araBAD promotor, coding for a SP_{BamD}-mScar-G_4S-BamD fusion protein under control of a Rha promotor
pSN143	BamA-mNeonGreen – BamD-mScarlet-I	KanR, pBBR1 ori, coding for a SP_{BamA}-BamA-G_4S-mNeon fusion protein under control of an araBAD promotor, coding for a SP_{BamD}-BamD-G_4S-mScar fusion protein under control of a Rha promotor
pSN144	BamA-mNeonGreen – mScarlet-I-BamD	KanR, pBBR1 ori, coding for a SP_{BamA}-BamA-G_4S-mNeon fusion protein under control of an araBAD promotor, coding for a SP_{BamD}-mScar-G_4S-BamD fusion protein under control of a Rha promotor
pSN149	BamA-mNeonGreen – mScarlet-I-$BamD^{E183A}$	Based on pSN144, coding for the $BamD^{E183A}$ variant
pSN150	BamA-mNeonGreen – mScarlet-I-$BamD^{Y184A}$	Based on pSN144, coding for the $BamD^{Y184A}$ variant
pSN151	BamA-mNeonGreen – mScarlet-I-$BamD^{Y185A}$	Based on pSN144, coding for the $BamD^{Y185A}$ variant
pSN152	BamA-mNeonGreen – mScarlet-I-$BamD^{E187A}$	Based on pSN144, coding for the $BamD^{E187A}$ variant
pSN180	BamA-mNeonGreen – mScarlet-I-$BamD^{R94A}$	Based on pSN144, coding for the $BamD^{R94A}$ variant
pSN181	BamA-mNeonGreen – mScarlet-I-$BamD^{R97A}$	Based on pSN144, coding for the $BamD^{R97A}$ variant
pSN182	BamA-mNeonGreen – mScarlet-I-$BamD^{L98A}$	Based on pSN144, coding for the $BamD^{L98A}$ variant
pSN183	BamA-mNeonGreen – mScarlet-I-$BamD^{N99A}$	Based on pSN144, coding for the $BamD^{N99A}$ variant
pSN293	BamA-mNeonGreen – mScarlet-I-$BamD^{V181A}$	Based on pSN144, coding for the $BamD^{V181A}$ variant
pSN294	BamA-mNeonGreen – mScarlet-I-$BamD^{Y184F}$	Based on pSN144, coding for the $BamD^{Y184F}$ variant

Table S2. Oligonucleotides used in this study

Name	Sequence	Orientation	Mutation
Pr347	TGGTAGTGTGGGGACTC	forward	n.a.
Pr1233	ATGGTGAGCAAGGGCGAGGAG	forward	n.a.
Pr1863	GCATGCATCGATCACCACAA	reverse	n.a.
Pr1878	GGTGGAGGTGGATCTATGGTGAGCAAGGGCGAG	forward	n.a.
Pr1880	GAGCTGTACAAGTAATTTGGCGGATGAGAGAAGATT	forward	n.a.
Pr1881	GGTTAATTCCTCCTGTTAGCCCA	reverse	n.a.
Pr1882	CAGGAGGAATTAACCATGGCGATGAAAAAGTTGCTC	forward	n.a.
Pr1883	AGATCCACCTCCACCCCAGGTTTTACCGATGTTAAACTG	reverse	n.a.
Pr1886	CAGGAGGAATTAACCATGGTGAGCAAGGGCGA	forward	n.a.
Pr1887	AGATCCACCTCCACCCTTGTACAGCTCGTCCATGC	reverse	n.a.
Pr1889	TTATGTATTGCTGCTGTTTGCG	reverse	n.a.
Pr1891	ATGTATATCTCCTTCTTAAGAATTGTTCATTACG	reverse	n.a.
Pr1892	TTACTTGTACAGCTCGTCCATGC	reverse	n.a.
Pr1895	GAGCTGTACAAGTAACTCGAGCCCCAAGGGC	forward	n.a.
Pr1896	GAAGGAGATATACATATGACGCGCATGAAATATCTGG	forward	n.a.
Pr1897	AGATCCACCTCCACCTGTATTGCTGCTGTTTGCGG	reverse	n.a.
Pr1899	TTACTTGTACAGCTCGTCCATGC	reverse	n.a.
Pr2118	ATGGTGAGCAAGGGCGAG	forward	n.a.
Pr2119	AGATCCACCTCCACCCTTGTACAGCTCGTCCATGCC	reverse	n.a.
Pr2120	GGTGGAGGTGGATCTTTCGTAGTGAAAGATATTCATTTCGAAGGC	forward	n.a.
Pr2121	GCCCTTGCTCACCATCCCTTCAGCACCGTATACGGT	reverse	n.a.
Pr2123	GGTGGAGGTGGATCTGTACCTGATAATCCGCCAAATGAAA	forward	n.a.
Pr2124	GCCCTTGCTCACCATTTCTTCCTTTGACCCCGAGC	reverse	n.a.
Pr2534	GTGATCGATGCATGCCTCACATTAATTGCGTTGCGC	reverse	n.a.
Pr2535	GTCCCCACACTACCATTAGCTCACTCATTAGGCACC	forward	n.a.
Oligonucleotides used for mutagenesis			
Pr2099	TCAGATGAAAGGTGCATGGCTGGGGA	forward	BamA^{E373K}
Pr2100	ATGCACCTTTCATCTGACGCATTTCGCGA	reverse	BamA^{E373K}
Pr2579	TATAGTACGCGGCCACGGAGTATTCATATTTCGC	forward	BamD^{E183A}
Pr2580	GTGGCCGCGTACTATACAGAACGTGGC	reverse	BamD^{E183A}
Pr2286	GCCGAGGCGTATACAGAACGTGG	forward	BamD^{Y184A}
Pr2287	TGTATACGCCTCGGCCACGGAG	reverse	BamD^{Y184A}
Pr2581	TTCTGTCGCGTACTCGGCCACGG	forward	BamD^{Y185A}
Pr2582	CGAGTACGCGACAGAACGTGGCGC	reverse	BamD^{Y185A}
Pr2583	CGCCACGCGCTGTATAGTACTCGGC	forward	BamD^{E187A}
Pr2584	TATACAGCGCGTGGCGCATGGGTTG	reverse	BamD^{E187A}
Pr3491	GCGAATAAACGCATCGATGGCAGCCTGTGC	forward	BamD^{R94A}
Pr3492	GCCATCGATGCGTTTATTCGCCTTAACCCGACC	reverse	BamD^{R94A}
Pr3493	CGGGTTAAGCGCAATAAAACGATCGATGGCAG	forward	BamD^{R97A}
Pr3494	CGTTTTATTGCGCTTAACCCGACCCATCCGAATATC	reverse	BamD^{R97A}
Pr3495	GGTCGGGTTCGCGCGAATAAAACGATCGATGG	forward	BamD^{L98A}
Pr3496	TTTATTCGCGCGAACCCGACCCATCCGAATATC	reverse	BamD^{L98A}
Pr3497	ATTCGCCTTGCGCCGACCCATCCGAATATC	forward	BamD^{N99A}
Pr3498	ATGGGTCGGCGCAAGGCGAATAAAACGATC	reverse	BamD^{N99A}
Pr3214	ACTCGGCCGCGGAGTATTCATATTTCG	forward	BamD^{V181A}
Pr3215	ATACTCCGCGGCCGAGTACTATACAGAAC	reverse	BamD^{V181A}
Pr3216	CTGTATAAAACTCGGCCACGGAGTATTC	forward	BamD^{Y184F}
Pr3217	GGCCGAGTTTTATACAGAACGTGGCG	reverse	BamD^{Y184F}

Supple. Text S1. Extended Materials and Methods

Plasmid construction

All plasmids are listed in Table S1. All oligonucleotides (primers) used in the construction of the plasmids are listed in Table S2.

mNeonGreen-mScarlet-I (pSN03): mNeonGreen was amplified from p15-mNeonGreen [38] with Pr1886 and Pr1887. mScarlet-I was amplified from mScarlet-I-PCNA [38] with Pr1878 and Pr1892. Pr1878 and Pr1887 also contained a sequence for a short flexible linker (GGGGS). The amplified fragments were fused by overlap-extension/splicing by overlap extension (SOE) PCR with Pr1892 and Pr1886. The backbone of pMATE-CelK [39] was amplified with Pr1880 and Pr1881. The mNeonGreen-mScarlet-I fragment was then cloned into the linearized backbone by InFusion™ cloning.

mScarlet-I-BamD (pSN08) and BamD-mScarlet-I (pSN09): bamD was amplified from chromosomal DNA of *E. coli* MG1655 with Pr1896 and Pr1889. The backbone of pMATE [40] was linearized with Pr1890 and Pr1891. The fragments were linked by InFusion™ cloning to generate the intermediate pBamD. This plasmid was then used to generate pSN08 and pSN09. For pSN08 mScarlet-I was amplified from mScarlet-I-PCNA [38] with Pr2118 and Pr1887. pBamD was linearized with Pr2123 and Pr2124. This way, mScarlet-I was inserted 3’ to the signal peptide of BamD, to avoid the cleavage of the fusion protein together with the signal peptide by the signal peptidase. Pr2123 and Pr1887 also contained a sequence for a short flexible linker (GGGGS). The linearized Backbone and mScarlet-I were combined by InFusion™ cloning. For pSN09 mScarlet-I was amplified from mScarlet-I-PCNA [38] with Pr1878 and Pr1899. pBamD was linearized with Pr1895 and Pr1897. Pr1878 and Pr1897 also contained a sequence for a short flexible linker (GGGGS). The linearized Backbone and mScarlet-I were combined by InFusion™ cloning.

mNeonGreen-BamA (pSN76) and BamA-mNeonGreen (pSN77): BamA was amplified from chromosomal DNA of *E. coli* MG1655 with Pr1882 and Pr1883. The backbone of pMATE-CelK [39] was linearized with Pr1881 and Pr1884. The fragments were linked by InFusion™ cloning to generate the intermediate pBamA. This plasmid was then used to generate pSN76 and pSN77. For pSN76 mNeonGreen was amplified from p15-mNeonGreen [39] with Pr1233 and Pr2119. pBamA was linearized with Pr2120 and Pr2121. This way, mNeonGreen was inserted 3’ to the signal peptide of BamA, to avoid the cleavage of the fusion protein together with the signal peptide by the signal peptidase. Pr2119 and Pr2120 also contained a sequence for a short flexible linker (GGGGS). The linearized Backbone and mNeonGreen were combined by InFusion™ cloning. For pSN77 mNeonGreen was amplified from p15-mNeonGreen [39] with Pr1878 and Pr1899. pBamA was linearized with Pr1880 and Pr1883. This way, mNeonGreen was inserted 3’ to the signal peptide of BamA, to avoid the cleavage of the fusion protein together with the signal peptide by the signal peptidase. Pr1878 and Pr1883 also contained a sequence for a short flexible linker (GGGGS). The linearized Backbone and mNeonGreen were combined by InFusion™ cloning.

mNeonGreen-BamA – BamD-mScarlet-I (pSN141): pSN76 was linearized with Pr2534 and Pr2535. The expression cassette of pSN08, starting from the rhamnose dependent promotor to the terminator was amplified Pr347 and Pr1863. The two fragments were combined by InFusion™ cloning.

mNeonGreen-BamA – mScarlet-I-BamD (pSN142): pSN76 was linearized with Pr2534 and Pr2535. The expression cassette of pSN09, starting from the rhamnose dependent promotor to the terminator was amplified Pr347 and Pr1863. The two fragments were combined by InFusion™ cloning.

BamA-mNeonGreen – BamD-mScarlet-I (pSN143): pSN77 was linearized with Pr2534 and Pr2535. The expression cassette of pSN08, starting from the rhamnose dependent promotor to the terminator was amplified Pr347 and Pr1863. The two fragments were combined by InFusion™ cloning.

BamA-mNeonGreen – mScarlet-I-BamD (pSN144): pSN77 was linearized with Pr2534 and Pr2535. The expression cassette of pSN09, starting from the rhamnose dependent promotor to the terminator was amplified Pr347 and Pr1863. The two fragments were combined by InFusion™ cloning.

Mutagenesis

Mutations were introduced to BamA or BamD by InFusion™ cloning using a single primer pair. The primers that were used are listed in Table S2. The plasmid used as a template for PCR is listed in the description of the plasmids in Table S1.

Calculation of spectral bleed-through

Calculations were done according to Hochreiter et al. [35]. In short, to correct the signal in the donor, acceptor and FRET channel for spectral bleed-through or crosstalk, spectral bleed-through factors were determined using two samples that only contained the donor fluorophore (BamA-mNeonGreen) or acceptor fluorophore (BamD-mScarlet-I). The spectral bleed-through factors allow the calculation of the corrected signals in the donor, acceptor and FRET channel.

$$S_1 = \frac{F_d}{D_d} \qquad \text{(S2)}$$

$$S_2 = \frac{F_a}{A_a} \qquad \text{(S3)}$$

$$S_3 = \frac{A_d}{D_d} \qquad \text{(S4)}$$

$$S_4 = \frac{D_a}{A_a} \qquad \text{(S5)}$$

$$D_{da}^c = \frac{D_{da} - S_4 * A_{da}}{1 - S_3 * S_4} \qquad \text{(S6)}$$

$$A_{da}^c = \frac{A_{da} - S_4 * D_{da}}{1 - S_3 * S_4} \qquad \text{(S7)}$$

$$F^c = F_{da} - D_{da}^c * S_1 - A_{da}^c * S_2 \qquad \text{(S8)}$$

S_1-S_4 are spectral bleed-through factors. Capital letters denote the channel in which the signal was measured (D = donor, A =acceptor, F = FRET). The small subscripted letter describes the fluorophores present in the measured sample (d =only donor, a = only acceptor, da = both donor and acceptor). A superscripted letter c denotes that the signal has been corrected for spectral bleed-through.

Calculation of normalization factors, relative donor and acceptor concentrations and DFRET values

The sample containing the fusion protein between mNeonGreen and mScarlet (mNeonGreen-mScarlet-I) was used to determine the normalization factors C1 and C2. The known FRET-efficiency was determined by spectral unmixing according to Alexeeva et al. [42] to be 0.43.

$$C1 = \frac{F^c - E * F^c}{E * D_{da}^C} \qquad \text{(S9)}$$

$$C2 = \frac{D_{da}^{c} * C1 + F^{c}}{A_{da}^{c}} \qquad \text{(S10)}$$

$$[don] = D_{da}^{c} * C1 + F^{c} \qquad \text{(S11)}$$

$$[acc] = A_{da}^{c} * C2 \qquad \text{(S12)}$$

$$DFRET = \frac{F^{c}}{C1 * D_{da}^{c} + F^{c}} \qquad \text{(S13)}$$

C1 and *C2* are correction factors to normalize the signal in different channels to each other. *[don]* and *[acc]* are the relative concentration of the donor and acceptor.

Calculation of the distance between the fluorophores of the FRET pair from the F_{max} value

The F_{max} value is representative of the FRET efficiency *E* of the interacting donor-acceptor pair. It can be used to estimate the distance between the acceptor and the donor.

$$E = \frac{1}{1 + (\frac{r}{R_0})^6} \qquad (14)$$

R_0 is the Förster distance of the specific FRET pair (in this case 61.48 Å). *r* is the actual distance between the fluorophores.
